# Recognizing Words and Reading Sentences with Microsecond Flash Displays

**DOI:** 10.1371/journal.pone.0145697

**Published:** 2016-01-22

**Authors:** Ernest Greene

**Affiliations:** Laboratory for Neurometric Research, Department of Psychology, University of Southern California, Los Angeles, California, 90089–1061, United States of America; University of Akron, UNITED STATES

## Abstract

Strings of dots can be used to construct easily identifiable letters, and these in turn can be used to write words and sentences. Prior work found that respondents could identify individual letters when all the dots were simultaneously flashed for an ultra-brief duration. Four of the experiments reported here constructed five-letter words with these dot-letters and a fifth experiment used them to write complete sentences. Respondents were able to recognize individual words that were displayed with a single, simultaneous ultra-brief flash of all the letters. Further, sentences could be efficiently read with a sequence of simultaneous flashes at a frequency that produced perceptual fusion. One experiment determined the frequency range that would produce flicker-fusion. Two experiments established the relation of intensity to probability of recognition with single flashes and with fused-flicker frequencies. Another established the intensities at which flicker-fused and steady displays were judged to be equal in brightness. The final experiment used those flicker-fused and steady intensities to display sentences. The two display conditions were read with equal efficiency, even though the flicker-fused displays provided light stimulation only 0.003% of the time.

## Introduction

“A word is obviously more complex than a letter, and efficiency for identifying the 26 most common three-letter words is 3%, a third that for letters, and efficiency for five-letter words is a fifth that for letters.” Denis G. Pelli [1]

In 1871 Rood [2] reported an observation that was difficult to believe and continues to challenge our understanding of visual mechanisms. He generated an electric spark that lasted less than a microsecond and claimed to be able to see the letters on a page from the light that it produced. There has always been some uncertainty as to whether this was a valid finding. There was little doubt that the spark itself was that brief. He used instrumentation and protocols for establishing flash duration that were developed by Wheatstone [3], which had produced a fairly accurate estimate of the speed of light [4]. Of greater concern was the question of whether he might have visualized a page that was in memory from seeing it in a lighted room.

Recent work from the present laboratory lends credibility to his claim, for not only can one see the presence of individual letters with a flash of light in the low microsecond range, but observers are able to name what letter was displayed by the ultra-brief flash [5]. This confirms the ability to register image information if flash intensity is sufficient. If multiple flashes are delivered at a frequency above 20 Hz, the flash sequence fuses and appears steady, *i*.*e*., as continuous emission. Further, at a certain ratio of intensities a steady display will appear comparable in brightness to a 24 Hz flash sequence that has perceptually fused, with either being adequate to provide for near perfect recognition of the letters [5]. Given that individual letters can be identified under these various conditions, one wonders whether words can be named when displayed in this manner.

There are numerous unanswered questions about the process by which briefly displayed stimuli can provide for visibility and memory access. With ultra-brief flashes in the low microsecond range the stimulus is over before the first change in membrane potential. Aside from the photochemical cascade, all of the neuronal signaling is post-stimulus, meaning that none of the information content is being delivered through sustained stimulus driving, *per se*. One assumes that self-generated sustained firing of one or more neuronal populations is providing for visibility of the stimulus that can last for about 100 milliseconds [6–9], and for persistent probing of memory that can last for 200 milliseconds or more [9–15].

Persistence of image information is pertinent, as well, when the stimuli are being delivered with sequential flashes. There is abundant evidence that a brief stimulus decays and becomes less effective at eliciting memory over time [9–15].

One might expect a steady display that delivers sustained image content to be more effective than an intermittent display that only provides decaying icons. Further, the time required to access memory may increase as the image becomes more complex, so a flash sequence that allows for letter recognition might not be sufficient for identification of words. These and related issues will be discussed once the experimental results have been presented.

**Fig 1:**
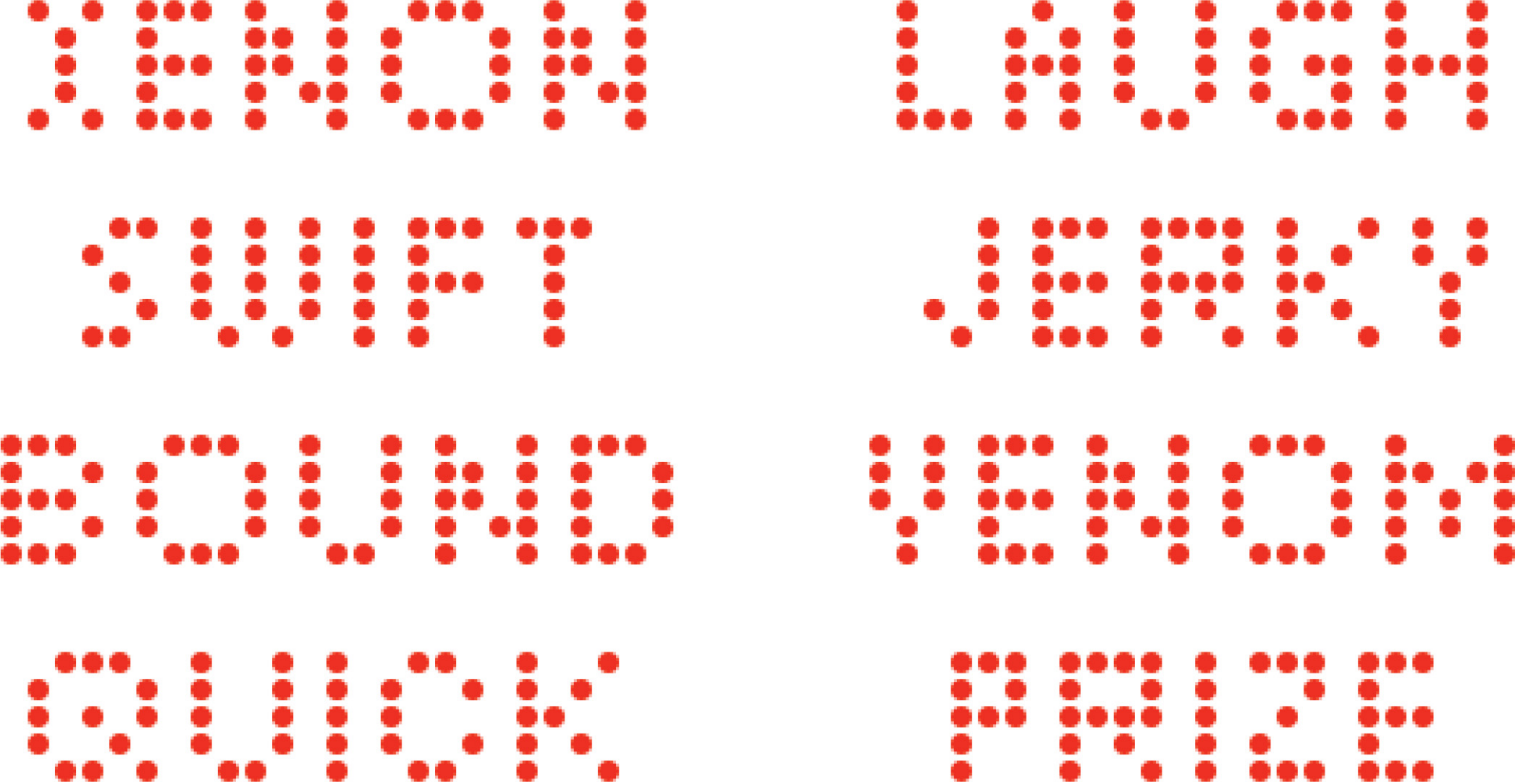
Examples of five-letter words rendered with dots. Example 5-letter words, from among the inventory of 320 words, show how the letters were formed using arrays of adjacent dots. Each letter of the alphabet is represented within these eight words. The words could be displayed with a single and simultaneous flash of all the dots in the word (Experiment 1), or with multiple flashes (Experiments 2–5).

The first four experiments examined recognition of five-letter words using the same basic protocol as the earlier work [5]. The words were displayed on an LED array wherein each letter of the word consisted of a pattern of discrete dots, as illustrated in Fig 1. The letters of the prior study [5] were formed as a relatively large and dense pattern of dots, but here a fairly minimal set of dots was used for each letter. The use of smaller letter patterns was to allow words and even sentences to be written on the LED array, and a fifth experiment examined the relative salience of fused-flicker and steady display of sentences.

## Methods

The experimental protocols for this project were approved by the University of Southern California Institutional Review Board. Respondents were recruited from the Psychology Subject Pool. Each respondent was provided with a written description of the task to be performed, and each signed an agreement to participate. A total of 40 undergraduate respondents were tested, eight in each of the five experiments– 27 females and 13 males.

In each of the following experiments, the term “flash” should be understood to mean a single and simultaneous pulse of light from each and all of the dots forming the letters of a given word. For the present report, being “ultra-brief” means that the duration of the pulse was just over one microsecond (see S1 Methods). Multiple flash sequences were specified according to the frequency at which the ultra-brief flashes were delivered. The term “steady display” describes a continuous emission of light from each and all of the dots forming the letters of the word or sentence. Each multiple-flash display of 5-letter words, whether with a flash sequence or with steady display, was for a duration of 750 milliseconds.

Flash and steady intensities were measured in radiometric units, specifically radiant intensity–microwatts or nanowatts per steradian. Details with respect to attributes and control of the LED display board, flash duration, intensities, task demands, and experimental protocols are provided in S1 Methods. The following is intended as an overview of the goals for each experiment.

The first experiment asked for recognition of words that were displayed using a single flash. Flash intensity was varied to derive an “intensity activation function” that specified how the probability of recognition (hit rate) changed as a function of intensity.

The second experiment displayed each word with a sequence of flashes, asking the respondent not only to say the word but also judge whether the sequence appeared to flicker. The frequency of the flash sequence was varied, and the main goal was to determine at what flash-rate the display would be perceived as fused, *i*.*e*., appearing as steady emission of light. At and above this frequency the stimulus sequence may be designated as a “fused-flicker” display.

The third experiment displayed the words using a frequency that was above the fusion threshold. Intensity was varied to determine how probability of recognition with fused-flicker displays changed as a function of intensity.

The fourth experiment displayed each word twice, once as an easily perceived fused-flicker display and also with a steady display wherein intensity was varied. Identification of the word was a task requirement, as in each of the prior experiments, but respondents were also asked to judge whether the two displays appeared to be equal in brightness. This provided a test of the classic Talbot-Plateau law [16–19], which asserts that the two conditions will be seen as equally bright when the average intensity of the fused-flicker display matches the intensity of the steady display.

A fifth and final experiment further assessed whether the two display conditions provided stimuli that were equally salient. An inventory of sentences was developed and displayed at the intensities that were judged to be most comparable in brightness in Experiment 4. The durations of the fused-flicker displays and steady displays were open ended, thus allowing the respondents to read the sentences at a comfortable pace. The time to read each sentence was timed with the goal of comparing readings times as a function of sentence length for the two display conditions.

For each of the first four experiments random effects semi-parametric logistic regression was used to model the treatment effects. Conceptually, a smoothly varying “average” curve is fitted for the probability of correct response over all participants, while the idiosyncratic deviations from the average response curve are incorporated using smooth person-specific random effects. This approach invokes only a minimal number of assumptions about the response curve–the main assumption is the lack of sudden jumps, and leads to fitted curves that follow the data closely. The downsides are that the parameter estimates are not meaningful, and the precision of the estimates is reduced compared to a well-fitting parametric curve. Technically, the average effect was modeled by a cubic spline with one or three* equally spaced internal knots, while the person-specific deviations were modeled with a random intercept and a penalized cubic spline which had two or three* equally spaced knots.

For Experiment 5 the relationship of reading time to sentence length was evaluated with a mixed effects model with fixed and random effects for condition, sentence length, and condition-by-length interaction. The model included a compound-symmetric covariance structure. Predicted values generated from this model were used to generate plots of the linear relationships between sentence length and reading time.

Reading time by sentence-length ratio data of Experiment 5 were evaluated with a mixed effect model for fixed and random effects, these being display condition (steady/fused-flicker) and respondents, respectively. The model also included a compound-symmetric covariance structure. For plotting purposed, the ratios were modeled using non-parametric kernel density estimates with a Gaussian basis function. Examination of residual error showed some tendency for non-normal error, with a bias toward larger positive error in shorter sentences. Alternative models adjusting for this tendency did not change conclusions regarding the difference between fused-flicker and steady display conditions, so the simpler results are shown. The sentences for which the reading was defective were not included in the main analysis described above. The proportion of error trials for fused-flicker versus steady treatments were submitted to logistic regression with random effects for respondents.

Analyses were performed using SAS version 9.3 (The SAS Institute, Cary, NC), using the Glimmix procedure for the primary analysis. The treatment effects in each of the first four experiments were found to be significant at p < 0.0001. Analyses of treatment effects in the fifth experiment were more elaborate, so significance levels are reported below.

## Results

### Hit Rate as a Function of Flash Intensity (Single Flash)

**Fig 2:**
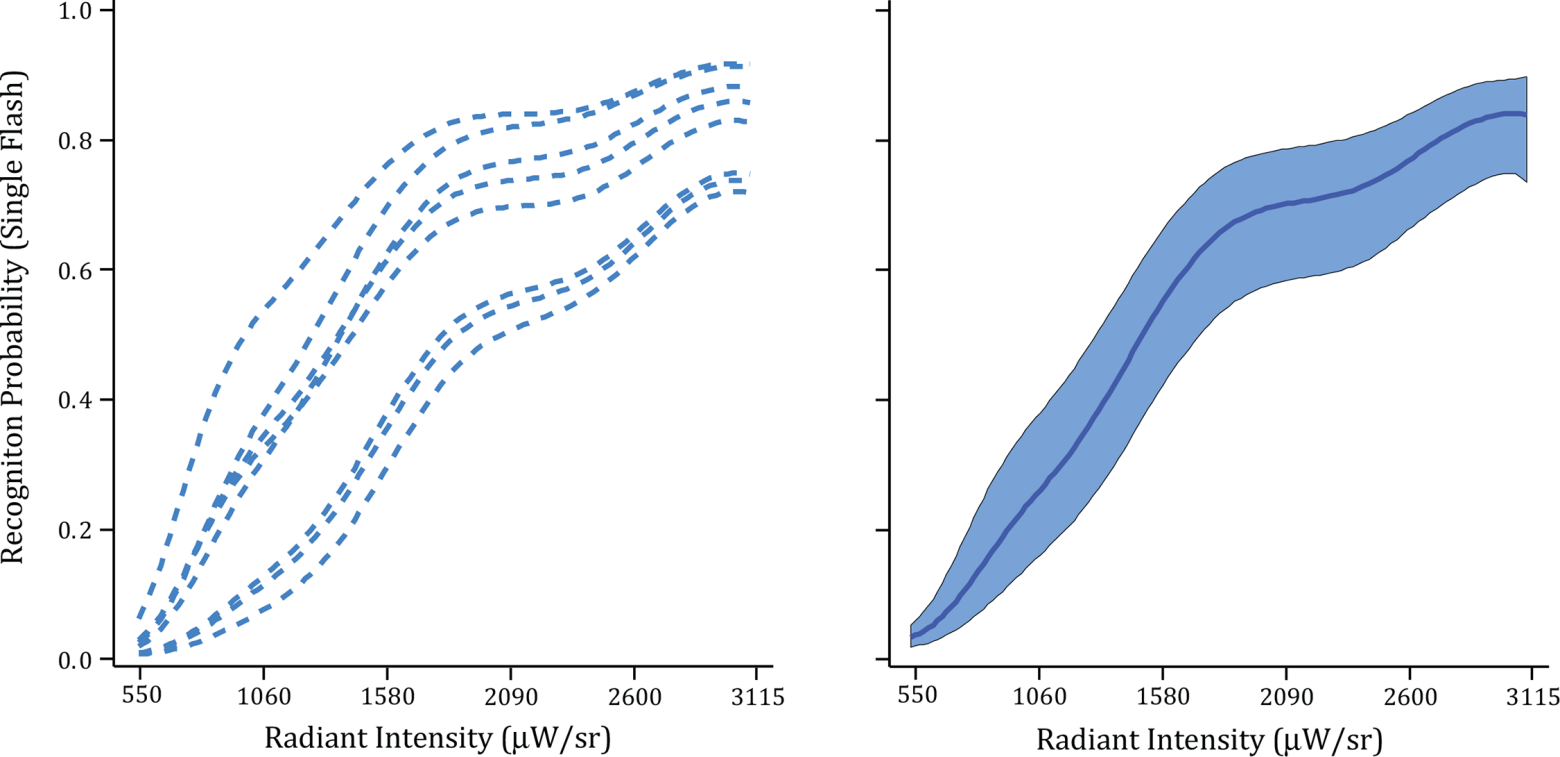
Word recognition probability with a single ultra-brief flash (Experiment 1). The plots show how the probability of recognition increased as a direct function of flash intensity. The left panel shows the models for the eight individual respondents. The right panel shows the group model, along with a 95% confidence band.

The two panels of Fig 2 show the results of Experiment 1, where each word was displayed with a single and simultaneous flash of all the dots forming the word and respondents were asked to report (name) the word that was displayed. Each model for the eight individual respondents as well as the group model manifested a monotonic rise in recognition with increased flash intensity, similar to what was found previously for recognition of individual letters [5]. However, flash intensity had to be much greater to provide a sufficiently salient word than was the case for individual letters. In the earlier work the rising portion of the activation curve for letter recognition was between 50 and 300 μW/sr [5], but here the intensity at the lower end of the word curve was twice that high and over ten times higher at the top. It seems most likely that the requirement for increased intensity can be attributed to the lean pattern of dots being used in the present experiment. Letters in the earlier experiment were relatively large and thick-bodied. The strokes for those letters were three dots wide whereas only a single string of dots was used for the strokes with the present inventory of letters. For each portion of the letter the lean configuration delivers less light per flash, so it is reasonable that intensity would need to be increased as compensation.

It is generally assumed that word recognition requires effective processing of the constituent letters [20,21]. An alternative possibility is that the pattern of contours within a well learned word is summarized as a single step, *i*.*e*., that shape encoding can be applied to multiple letters without registering each individually. Research showing “word superiority effect” suggests that possibility.

Reicher [22] and Wheeler [23] provided the classical “Reicher-Wheeler paradigm,” wherein words, non-words, and/or single letters were presented briefly and then masked, followed by force choice among alternatives. For example, Reicher [22] briefly displayed a word such as WILD, a nonword anagram of the word, such as DILW, or a single letter by itself, *e*.*g*., L. This was followed by a visual mask, then by two alternative target letters, *e*.*g*., L and N. Both of the alternative choices would form a word, in this case WILD and WIND. The respondents were asked to say which of the two letters had been presented prior to the mask. They consistently performed better if the initial stimulus was a word rather than a non-word, and even relative to a letter that was presented n isolation.

The words enjoy a processing advantage compared to single letters, apparently due to top-down facilitation. Starrfelt and associates [24] have provided an interesting advance on this issue. They studied recognition of 3-letter words and single letters, and found that reaction times were significantly shorter for words than for letters. A second experiment examined display of the letters and words for intervals ranging from 6 to 80 ms, terminated by a pattern mask composed of letter fragments. Words were identified significantly better than letters for all exposures from 19–37 ms, with other durations manifesting ceiling and floor effects.

The present data does not directly address whether one must register the individual letters of a word as a first step toward identifying the word that was displayed. However, the results do indicate that it is not necessary to provide a protracted display of the word so as to allow for successive sampling of letter content. A flash duration in the low microsecond range is sufficient to elicit word-encoding operations.

### Flicker Fusion with Multiple Ultra-brief Flashes

The second experiment tested 8 new respondents, displaying the 5-letter words with multiple ultra-brief flashes and providing the flash sequence for 750 ms at frequencies ranging from 6 to 30 Hz. The intensity of flashes was set at the mean level at which each respondent in the first experiment first reached a maximum hit rate. This level of intensity assured a salient stimulus that could be readily identified, and the mean hit rate for recognition for respondents in this experiment was 0.973.

**Fig 3:**
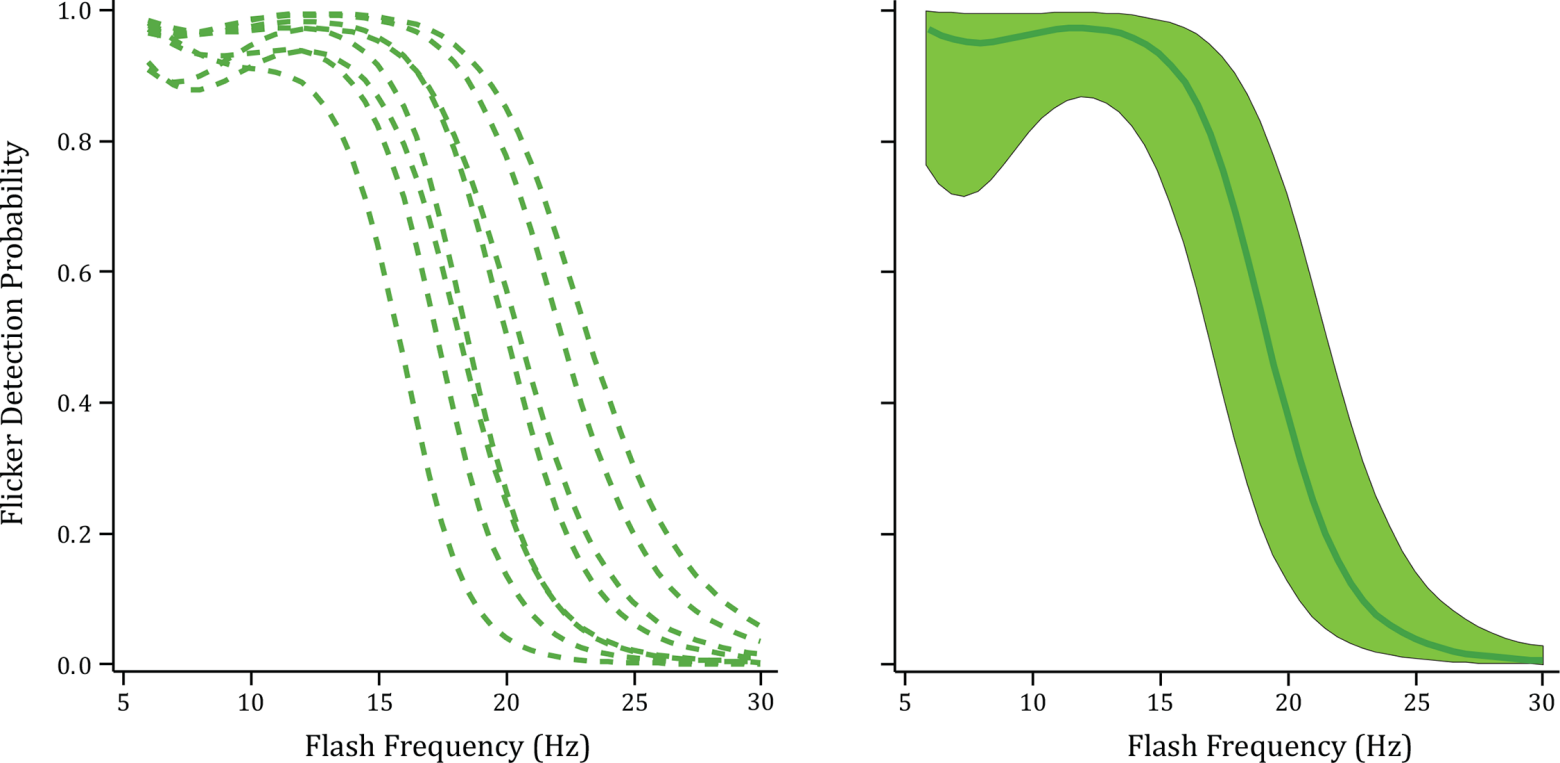
Flicker fusion probability as a function of flash frequency (Experiment 2). The individual respondent models (left panel) and group model (right panel) reflect the probability of flicker detection as a function of flash frequency. Each flash was at an intensity that produced a high hit rate in Experiment 1. All respondents manifested a progressive decline in flicker detection as flash frequency was increased.

Respondents were also asked to indicate whether the display appeared to flicker. Fig 3 shows the probability that flicker was detected at the various display frequencies. Every respondent manifested a monotonic decline in flicker detection as the frequency of flashes was increased. The decline began for the group mean at around 13 Hz and reached chance levels in the 27–28 Hz range. The classic critical flicker threshold is defined as the frequency at which flicker detection is at 50%. For the present data the mean was at 19 Hz, with the range across respondents being 16 to 23 Hz.

The flicker detection functions for words were shifted toward higher frequencies than was found for letters tested with similar task conditions [5]. In the earlier study the group mean for letters began its decline at around 10 Hz (words being 13 Hz) and was at chance by about 20 Hz (versus 27–28 Hz). The critical flicker threshold for letters was 14 Hz (words being 19 Hz). Undoubtedly the higher values for words was due to the use of higher intensity flashes, for it is well established that flicker is detected at progressively higher frequencies as intensity is increased [25].

The flashes provided by the LED array have a narrow wavelength spectrum centered on 630 nm (red). The room illumination was mesopic (dim) which would allow activation of rods if the stimulus were not too bright and the wavelengths extended well into the blue-green range. However, given the intensity and wavelength range of the present displays, it is unlikely that rods or blue cones were activated, and at most the influence on green cones would be fairly weak. In keeping with earlier findings by Hamer & Tyler [26], it seems likely that red cones generated the slgnal that allowed perception of flicker.

Given the flash intensity, a fusion-flicker threshold of 19 Hz is rather low relative to prior reports [25, 27–30], but this threshold is fairly consistent with the earlier work where letters were displayed [5]. The relatively low critical fusion frequency may be due to the use of an ultra-brief flash duration. The flash occupies a small fraction of each period within the flash sequence with the remainder of the period being dark. Most of the prior work on flicker fusion has used a 50% duty cycle, *i*.*e*., emission for half of each period and dark for the remaining half. A number of prior reports have found that fusion takes place at lower intensities as the duty cycle is reduced [31–37].

### Varying Intensity with Fused-Flicker Displays

Experiment 1 provided a single-flash activation curve, *i*.*e*., a function showing the probability of recognition as a function of flash intensity. The goal of Experiment 3 was to provide a multiple-flash sequence that was perceived as steady and determine the intensities that would yield a fused-flicker activation curve for word recognition. The earlier experiment with letters [5]] used a 24 Hz sequence, this being above the frequency at which respondents reliably saw the stimulus as steady emission. Here the group model was above zero at 24 Hz (see Fig 3), but a vast majority of the displays were judged as steady by each of the respondents. Further, pilot work suggested that some respondents saw flicker at this frequency if they were charged with looking for flicker, and saw none if they were not being asked in advance to make this judgment. Given this, it seemed reasonable to keep the protocol consistent with what had been done with letters; therefore a 24 Hz frequency was used for display of multiple flashes in Experiment 3.

**Fig 4:**
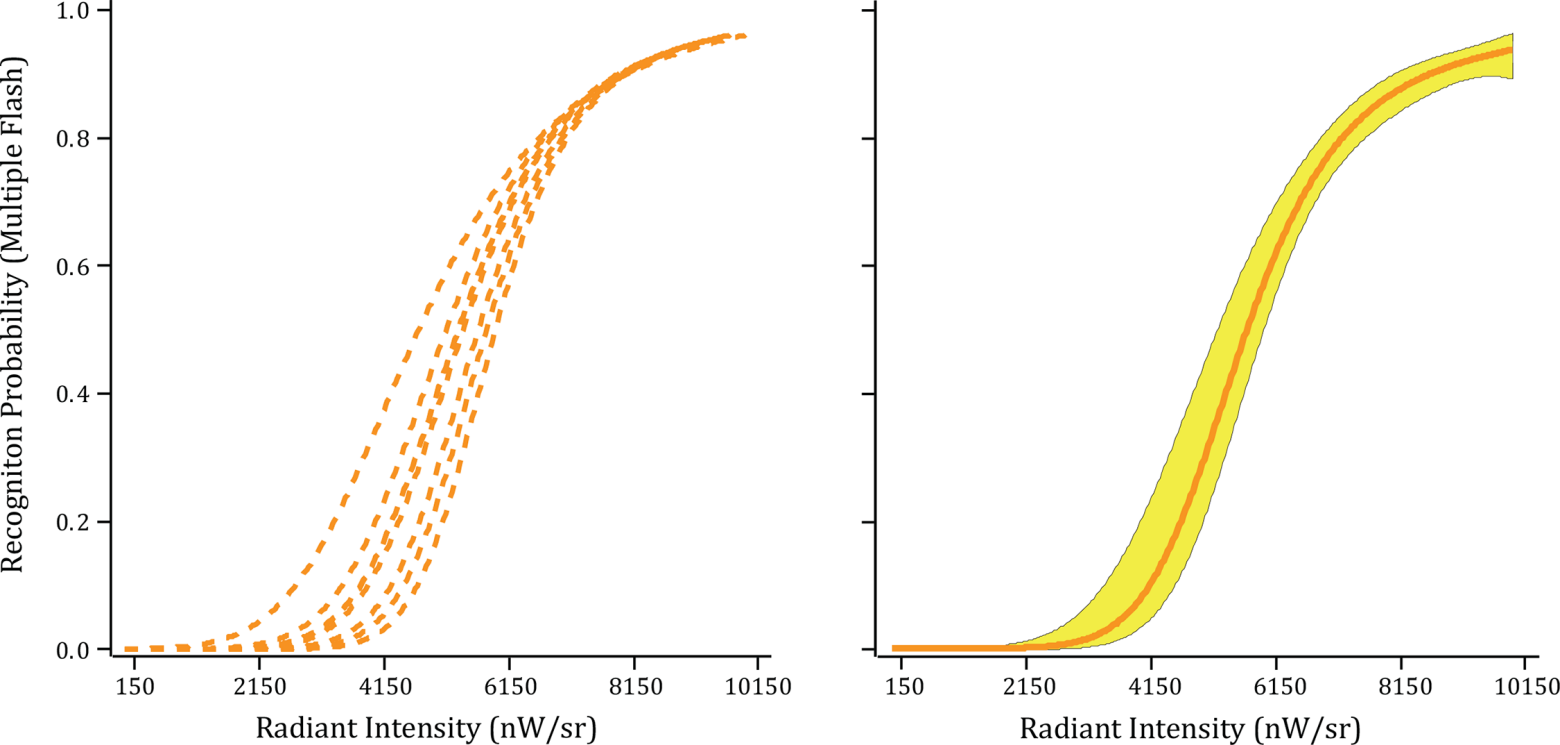
Hit rate as a function of steady emission intensity (Experiment 3). The individual respondent models (left panel) and group model (right panel) reflect changes in the probability of recognition with changes in the intensity of a 24 Hz flash sequence of ultra-brief flashes. The results demonstrate that far less intensity is needed to produce a given hit rate if the stimulus is displayed with a sequence of flashes.

The inventory of words was shown to each of the new respondents, displaying the words with a sequence of ultra-brief flashes at 24 Hz for 750 ms and varying the intensity of the flashes. The models for each respondent rose monotonically from below threshold to near perfect recognition, as shown in Fig 4. Five of the eight respondents reached perfect recognition at the high end of the intensity range, with each of the other three making only a single recognition error.

As expected, the use of multiple flashes produced a drop in the intensity needed for recognition, the difference being over two orders of magnitude. Finding that far less intensity is needed with multiple flashes affirms the concept that the impact builds in strength rather than simply providing protracted activation support. Prior research from this lab has shown that a near-threshold intensity that is ineffective when delivered as a single flash can elicit high levels of shape or letter recognition if the stimulus is shown again with a second flash at the same intensity [26,27]. The present results confirm the concept that salience of the stimulus is determined by net energy. This is specified by the Talbot-Plateau law, which will be discussed subsequently.

### Brightness Equivalence of Fused-Flicker and Steady Displays

Experiment 4 displayed each word in the inventory in two ways, once as a sequence of ultra-brief flashes at 24 Hz for a duration of 750 ms, and also as steady emission from the dots forming the word for 750 ms. The flash sequence used an intensity that was the mean at which each of the respondents in Experiment 1 first reached his or her highest hit rate. The intensity of the steady displays was varied across a range that was expected to be perceived as less bright than the flash sequence at the low end of the range and brighter than the sequence at the high end of the range. Respondents were asked to name the words and also say whether the two displays appeared equally bright (scored as 1) or different in brightness (scored as 0). The expectation was that resulting models would be shaped as an inverted U, with the peak reflecting the intensity at which the two displays were seen as most equal in brightness.

With words being displayed twice for an extended duration, *i*.*e*., 750 ms, a high level of recognition was expected and was found. Three of the respondents were able to name all of the words, and the other five scored a 0.99 hit rate.

**Fig 5:**
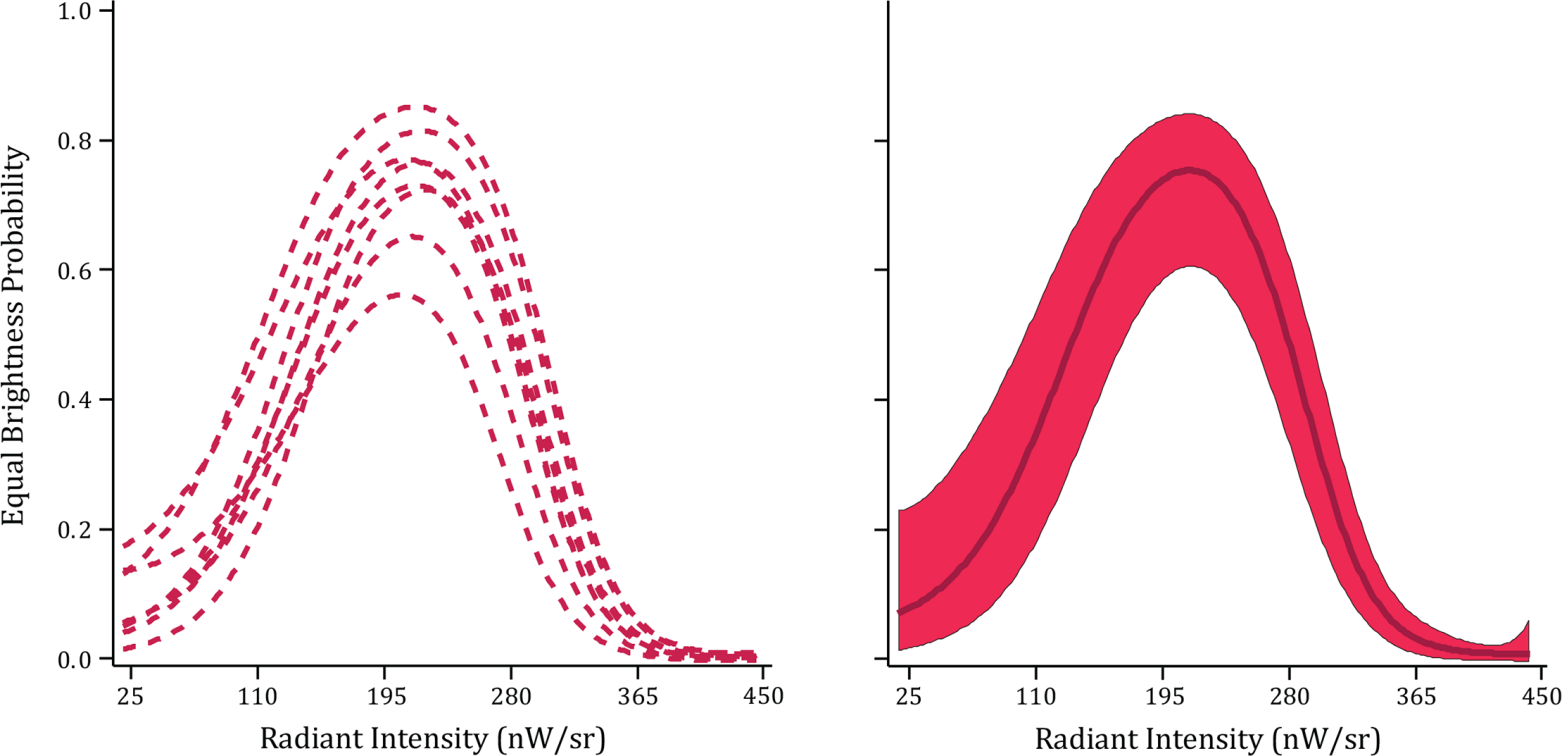
Equating for brightness of fused-flicker and steady displays (Experiment 4). Words were displayed twice, once as a sequence of ultra-brief flashes at 24 Hz (750 ms) with the intensity fixed, and also with steady emission for a 750 ms interval with intensity being varied. Respondents were asked to judge whether the two displays appeared equal in brightness. The model for each respondent (left panel) and the group model (right panel) show the intensity at which the steady display is judged to be most equivalent to the sequence of ultra-brief flashes.

The responses of interest for this experiment were the brightness judgments. The models shown in Fig 5 confirmed the expectation–each was an inverted U-shaped function. The recognition probability of the peaks varied from less than 0.6 to over 0.8, but the models peaked at very close to the same display intensity for all of the respondents.

Where respondents found brightness to be most equal, the flashes were being delivered at 2573 μW/sr and the steady display was at 0.102 μW/sr. The two intensities differ by more than five orders of magnitude, as was the case where letters were seen as comparable in brightness [5].

Talbot [16] and Plateau [17] provided early evidence that the perceived brightness of a flash sequence will be judged as equal to a steady display when the average intensity of the former equals the intensity of the latter. Calculating the average intensity means that one includes the dark portion of the display as having zero intensity, this being weighted by the time during which the display is dark. The measured duration of flashes was about 1.3 μs, so the average intensity of the flash sequence is the intensity multiplied by the duration and number of flashes, then divided by the 750 ms display period. This calculation yields an average intensity of the fused-flash sequence of 0.086 μW/sr, a value that is remarkably close to the steady intensity of 0.102 μW/sr.

The difference between the steady intensity and the fused-flash average intensity, calculated prior to rounding, is 17%. This could mean that flashed stimuli are more salient due to the Broca-Sulzer [38] effect, which finds an enhancement of brightness with short-duration flashes [30–43]. The earlier study that examined recognition of letters found that steady emission intensity was about twice as large as the average intensity of a 24 Hz flash sequence. It is hard to know whether this is a meaningful difference, for the use of such a large denominator in the calculation of average intensity– 750,000 microseconds, can magnify and potentially distort small differences in measured values.

Even where the intensity of a steady stimulus is twice the average intensity of a flash sequence, the results of the two studies are amazingly close to what is specified by the Talbot-Plateau law. This principle was formulated on the basis of a 50% duty cycle, *i*.*e*., where the light and dark portions of the cycle are of equal duration. Here, as with the earlier study using letters [5], one is dealing with ultra-brief flashes where a substantial portion of the cycle is dark.

It appears that the Talbot-Plateau law has substantial validity even where flash durations are in the microsecond range and thus the light and dark intervals are dramatically different.

### Sentences Displayed with Fused-Flicker and Steady Emission

The prior experiments demonstrate that 5-letter words can be reliably identified when displayed with ultra-brief flashes. Also, if the average intensity of a flash sequence is roughly the same as the intensity of a steady display, the two display conditions will be judged as being equal in brightness. Brightness may well determine the salience of a given display, serving as an index of how effective that display will be for eliciting recognition. However, it would be good to have additional evidence that a fused-flicker display allows for effective processing of verbal material. Experiment 5 evaluated this matter by having respondents read complete sentences that were presented as steady or fused-flicker displays, and recording the time required to read each sentence.

**Fig 6:**
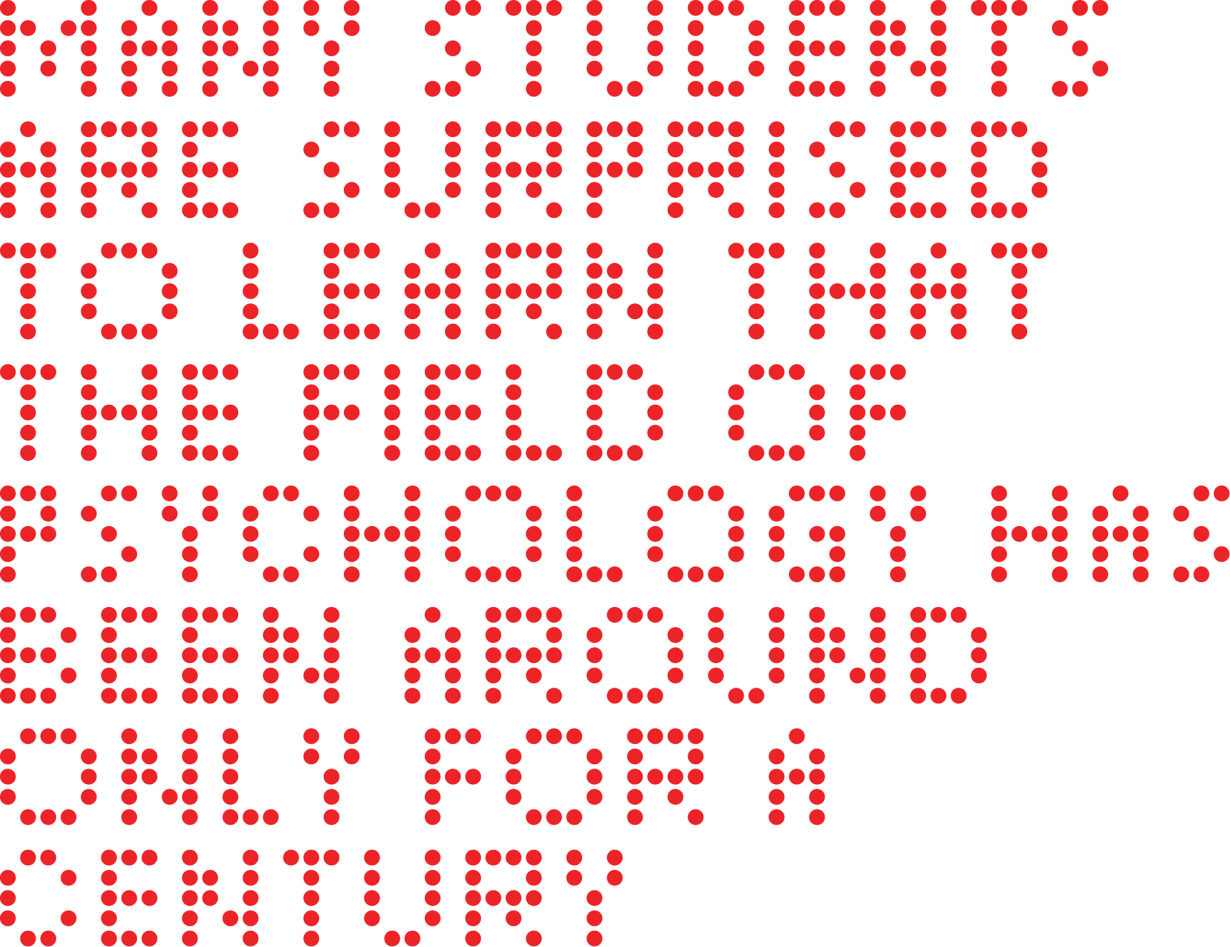
Example of a sentence displayed with fused-flicker or steady emission (Experiment 5). A typical sentence is shown as it would appear on the LED display board. The display would either be with steady emission of light from all the dots or as a sequence of flashes that had fused to appear steady. Respondents were asked to carefully read the sentences with the expectation that any differentials of reading time would indicate the relative salience of the display method.

Experiment 5 drew from an inventory of sentences that were written with the thin dot-letters that were used for 5-letter words. Fig 6 provides an example of how one of the sentences would be structured for display on the LED board.

The sentences were displayed with either a fused-flicker sequence of flashes or steady emission, using intensities that were judged to be equivalent in brightness in Experiment 4. Each sentence was shown continuously as long as needed for the respondent to read the sentence. The measures of reading time as a function of sentence length, display treatments, and respondents were evaluated using a mixed effects model (see Methods). A mixed effects model found sentence length to be significant, *i*.*e*., p < 0.0001, the same as for earlier experiments. Least-square mean estimates for display conditions are given in Table 1. Display condition and the interaction of condition by sentence length were not significant (p = 0.0578, 0.9090, respectively). The random respondent effect was strongly significant (p < 0.0001). Removing the interaction term from the mixed model, the estimated overall slope was 87.98 (SE = 7.53, p < 0.0001).

**Table 1:** Analysis of sentence reading time (Experiment 5). Least-square mean estimates for fused-flicker versus steady display conditions did not suggest any difference in reading time.

Condition at average sentence length	Reading Time Estimate (ms)	Standard Error	p-value	Confidence Bounds	
				Lower	Upper
Fused-flicker	7084	436	---	6149	8019
Steady	7280	436	---	6346	8215
Difference (FF-S)	-196.5	95.1	0.0578	-400.4	7.45

**Fig 7:**
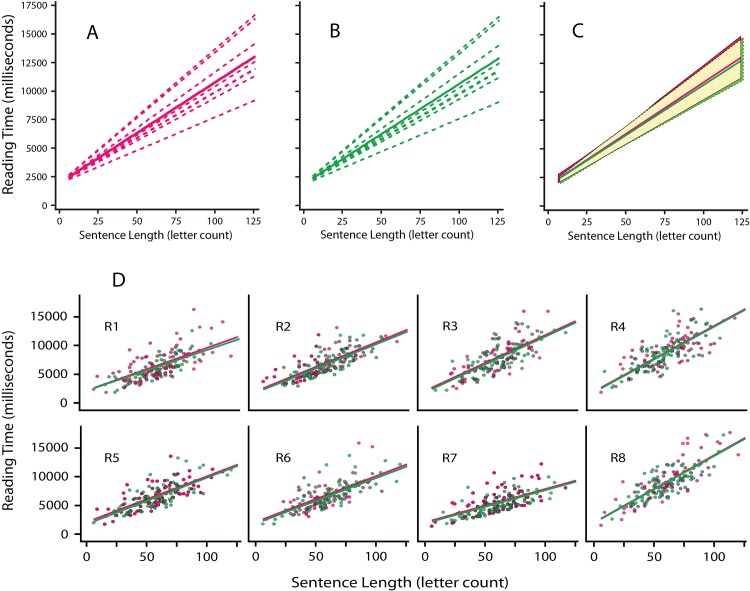
Regression models for sentence reading time (Experiment 5). A and B. These panels show linear regression models for sentences that were read with steady emission of light (red) or with fused-flicker displays (green); each model shows reading time as a function of sentence length. Dashed lines show the models for individual respondents, and the solid lines are the models for the group. C. The group models for steady and fused flicker displays are almost completely overlapped–red for steady and green for fused-flicker. The confidence bands for each model also overlap almost completely, the zone of the overlap being colored in yellow. To help distinguish each zone, the confidence interval for the steady and fused-flicker displays are bounded with solid and broken lines, respectively. Vertical spans of the confidence intervals are accurate, but the range of each interval has been extended a bit to better delineate them. D. Each panel shows plot-points for each sentence, *i*.*e*., reading time as a function of sentence length, and also shows the linear regression models for the two conditions. Tokens as well as the models are red and green for sentences displayed with steady and fused-flicker emission, respectively. The eight respondents differed in terms of the slope of the regression models, but in each case the steady and fused-flicker displays produced models that are almost identical. One may need to magnify the panels to see the red and green lines for each respondent.

Predicted values generated from the mixed effect models were used to generate plots of the linear relationship between sentence length and reading time. Fig 7 shows these models for individual respondents and for the group with respect to each of the treatment conditions, *i*.*e*., steady and fused-flicker displays. Individual respondents read at different rates, which is reflected in the slope differentials shown in Fig 7A and 7B. The group models shown in Fig 7C are almost identical in terms of slope as well as overlap of the confidence intervals for the two display conditions.

Fig 7D plots the individual models as separate panels showing also the scatterplots of raw data from which each model was derived. The reading times for steady displays are shown with red dots and green dots are used for the fused-flicker data. The models for these two display conditions are also shown with red and green plot-lines, but those models are almost completely overlapped, so magnification may be needed to see the two lines.

The proportion of misread sentence during the test session (data not included in the analysis described above) was less than 8%. The overall error rates for fused-flicker and steady-emission displays were 7.79% and 7.78%, respectively. Individual respondent error rates ranged from 0 to 11.1%. A logistic regression with random effect for respondents found no significant difference in error rates for the two display conditions (Odds ratio = 1.02; 95% confidence interval = 0.66, 1.57; p = 0.9218.

To further examine the relative effectiveness of steady and fused-flicker displays, a ratio was calculated for reading time divided by sentence length, which can be described as “reading efficiency.” These data were analyzed used a mixed effects model with fixed and random effects for display condition and respondents, respectively. There was very little difference in the influence of the two display conditions (p = 0.5219). Least-square mean estimates of the display-condition ratios are given in Table 2. Respondents differed significantly (p < 0.0001).

**Table 2:** Analysis of reading efficiency (Experiment 5). There was no statistical evidence of a difference in reading efficiency ratios for fused-flicker and steady displays.

Condition	Reading Efficiency Ratio Estimate	Standard Error	p-value	Confidence Bounds	
				Lower	Upper
Fused-flicker	124.75	7.78	---	108.05	141.44
Steady	131.98	7.78	---	115.28	148.67
Difference (FF-S)	-7.23	11.01	0.5219	-30.84	16.38

**Fig 8:**
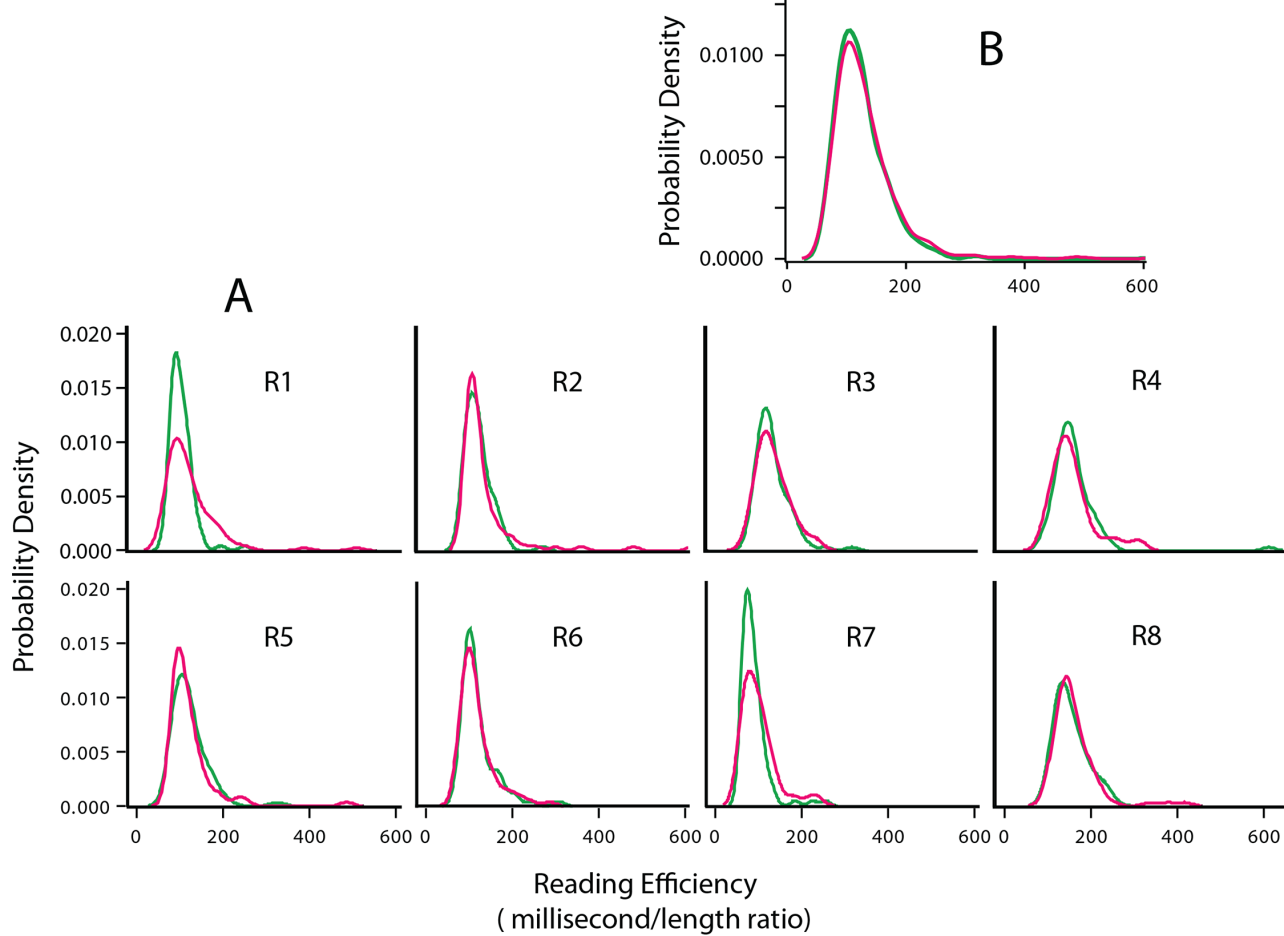
Modeling reading efficiency (Experiment 5). An alternative way to illustrate relative salience of the two display methods is to form a ratio of the reading time over sentence length. The distribution of sentence lengths was very close to a normal curve, therefore kernel estimates derived from these ratios, designated as reading efficiency, would be expected to have similar shapes. This was confirmed in the estimates for individual respondents as well as the group estimates, shown at A and B, respectively. There was substantial overlap of individual kernel estimates, and the group estimates for steady and fused-flicker displays are almost identical. Red = steady sentence display; green = fused-flicker sentence display.

The inventory of sentences had lengths that approximated a Gaussian distribution (see S1 Methods), and reading time had been shown to be a linear function of sentence length (see above). Therefore the intuition was that kernel estimates that were used to model the reading efficiency ratio would be approximately Gaussian in shape. This was confirmed as can be seen in the panels of Fig 8. Fig 8A shows the steady and fused-flicker reading efficiency kernel estimates for individual respondents. Here each respondent manifests a small differential in overlap of the kernel estimates, but in general one can see that the treatment effects are very similar. Fig 8B shows the kernel estimates for the group, *i*.*e*., averaged across respondents. The plotted estimates are almost identical.

The results of Experiment 5 affirm that the two display conditions provide for equivalent reading efficiency. The intensity used for the fused-flicker and steady conditions were the ones that respondents of Experiment 4 judged to be most comparable in brightness. Combining these results supports the conclusion that a sequence of 24 flashes *per se*cond, each flash being only a bit longer than one microsecond, can produce a stimulus that is perceived as steady, is equal in brightness, and is equally salient for providing readable sentences. This is the case even though light is being delivered for only 31.2 microseconds out of every million microseconds, a proportion that is less than 0.003%.

The present experiments were not designed to evaluate any particular theory of word or sentence processing, but it may be useful to review some of this work. As mentioned above, if one displays a written stimulus that is degraded by noise or brief presentation, letters in words are reported more accurately than are single letters or letters that are embedded in non-words. This has been described as word superiority effect [22,23,44]. The Reicher-Wheeler paradigm used words, non-words, and single letters presented for a single brief exposure, followed by forced-choice decisions for which of two letters was shown The letter options were identified more accurately if they were embedded in words. McClelland & Rumelhart [45] explained this with a model wherein feedback connections from higher processing levels served to strengthen or inhibit lower-level processing.

Pelli and associates [20] provided a model of word perception wherein letters are perceived independently with separate detection decisions for each letter. The model extracted measures of reading efficiency from the time required to read words of different lengths. Reading accuracy depended on roughly the same amount of per-letter information as the number of letters increased, suggesting that each letter contributed independently to recognition. This can be described as an independent parallel processing model, somewhat akin to those proposed by Massaro [46] and Estes [47].

To assess the question of whether the letters are independently processed, Houpt and associates [48] derived a “capacity coefficient”–a response-time measure of processing efficiency (see also [49–51]). Reaction times for words were faster than predicted by the parallel-processing baseline, which provided evidence for a special benefit for reading words, *i*.*e*., a facilitation of capacity.

Starrfelt and associates [24] found word superiority over single letters in vocal reaction times across a range of exposure durations from 6 to 80 milliseconds. They infer that words are processed better and faster than letters from threshold of perceptibility. This supports the assumption that letters rely more on bottom-up processing, whereas words receive some benefit from top-down mechanisms. As an interesting complication, they found the opposite when several items were simultaneously displayed, suggesting that the word-processing mechanisms may become overloaded. From this they infer that the words are not treated as whole units by visual short-term memory.

There is considerable interest in the neural substrates for processing of visual language information. In 1892 Dejerine [52] described a patient with a lesion in the left occipito-temporal junction that produced a selective deficit for reading letters and words. This was the first evidence of a putative visual word-form area (see [53] for a review). Gaillard and associates [54] describe a patient in whom this region was resected to deal with seizures, who then could only manage to read words in a slow letter-by-letter fashion, though object and face recognition was unaffected. Similar findings of letter-by-letter reading after left occipito-temporal brain injury have been reported by several other groups [55–58]. These patients have normal language functions, including good recognition of single letters, but show substantial impairment in processing of letter strings. It is possible that single letters are processed in a slightly different region than are words and letter strings [59–60].

Neuroimaging work in normal readers has shown that the left fusiform (occipito-temporal) gyrus responds more strongly to words and pseudowords than to strings of consonant letter strings or non-linguistic characters [61–64]. Some studies show that the orthographic system uses a summary code that is unaffected by changes in letter case [62,64,65]. Kronbichler and associates [66,67] has hypothesized that this region responds to whole-word orthographic forms, and is especially sensitive to orthographic familiarity. The findings of Bruno and associates [68] supports this proposition. Using a Postman protocol [69], Binder and associates [70] quantified the degree of orthographic approximation to normal English words, and found a significant increase in fMRI signal strength in the left fusiform gyrus as a function of letter-sequence probability. No other brain region manifested this response pattern.

Cohen and associates [71,72] have formulated a combinatoric detection model for reading that has levels that are progressively tuned for letter fragments, individual letters, letter combinations, and whole words using classic neural network principles. They further hypothesize that the hierarchical structure of the model is topographical organized on the visual word-form area along its anterior to posterior axis. To test this hypothesis, they exposed adult readers to stimuli that included false-font strings, strings of infrequent letters, strings of frequent letters, strings of frequent quadigrams, and real words [72]. They found a gradient of selective fMRI activation throughout the occipito-temporal cortex, with activation becoming more selective for higher-level stimuli toward the anterior fusiform word-form system.

## Discussion

Prior research from the present laboratory has found that a single ultra-brief flash can elicit recognition of diverse shapes, including letters [5,9,73]. The present research demonstrates that a flash that is little over one microsecond in duration can elicit recognition of five letter words. Further, full sentences that were displayed with a fused-flicker sequence of ultra-brief flashes were read with the same ease and efficiency as were sentences displayed with steady light emission. It was far from obvious that the flash displays would provide these findings. Notwithstanding the word superiority effect discussed above, Denis Pelli and associates [20] have provided suggestive evidence that three-letter words are identified with lower efficiency than are discrete letters, and the efficiency for five-letter words is lower yet. Their task provided degraded stimuli for two hundred milliseconds. Although these were low-contrast stimuli, at least the visual system was provided with extended light exposure, as might be needed to register the patterns and filter out stimulus noise. The ultra-brief flashes used in the present work would need to launch sustained activity within the retina and subsequent visual processing stages, and there was no assurance that the degree and duration of activation would be sufficient to elicit recognition or allow efficient reading.

We now have confirmation that the activation provided by the ultra-brief flash does provide for recognition of five letter words and efficiency of sentence reading is no different whether one is displaying the words with a fused sequence of these flashes or with steady emission. Let’s now discuss the neural and perceptual mechanisms by which flashes produce effective neuronal activation.

There is evidence that flash-elicited recognition is possible because of information persistence. Recognition of shapes and letters is minimal if the stimulus is shown with a very brief flash at near-threshold levels of intensity. However, providing a second flash at the same intensity can boost recognition into a high (or maximum) range, and this summation of influence declines to zero in about 100 ms [9,73]. Similar results have been found in other two-pulse detection experiments [6,74,75]. Masking and related protocols show similar declines in persistence of stimulus information [76,77]. The flash likely generates some form of persistent neural activity that lasts far longer than the flash duration, *per se*, this time being needed for shape encoding. Further, such persistence would have a clear role for providing perceptual fusion of a flash sequence.

One potential source of this persistence is the impulse response of photoreceptors, both rods and cones, though here we focus on cone responses. Across a number of species, a very brief flash will elicit a cascade of photochemical reactions that builds to a peak over many tens of milliseconds and then slowly declines across a much larger interval [78–85].

The issue becomes more complicated because of reports that the cones of primates (including humans) show biphasic impulse responses [86–90]. The findings of Schnapf and associates [89] are representative. They found that the photocurrent generated by an 11 ms flash has an initial component that peaks in about 50 ms and lasts for about 100 ms (depending on intensity of the flash), with a reverse component (described as inhibitory) that lasts for upward of 250 ms before returning to zero. The second component of red cones had lower amplitudes and were somewhat shorter than those of green and blue cones.

Some human perceptual judgments may be derived from biphasic impulse responses. Blackwell [91] appears to be the first to observe inhibitory effects with two-pulse task, finding that threshold detection was facilitated if the two pulses were delivered within a very short interval, with suppression of detection at a somewhat longer interval. Ikeda [92] followed up with supporting data and models that manifested excitatory and inhibitory components. Others have reported similar results [93,94]. Burr & Morrone [95] reported evidence for biphasic impulse responses for achromatic summation but not for chromatic summation.

However, tests of threshold detection may not pertain to the persistence of complex image information, especially at the higher photon densities required for recognition of shapes. Prior results from this laboratory found monotonic declines in recognition with two-pulse method where the pulses consist of shapes and letters that were displayed with ultra-brief flashes [9,73]. It is possible that shape-encoding mechanisms register the initial impulse component and are not influenced by the inhibitory phase that follows.

As an alternative basis for monophasic declines in information persistence, there is evidence that the basic impulse response of primate cones may be monophasic. Human electroretinograms and modeling suggest this possibility [96,97]. For example, van Hateren & Lamb [97] found that the human cone response is monophasic and faster than previously reported–peaking in about 20 ms. Further, Cao and associates [98] found mostly monophasic responses from each cone class using patch-clamp recordings in macaque. Low intensity flashes produced impulse responses that returned to zero in about 100 ms, with the duration of the response being progressively longer as flash intensity was increased. These investigators suggest that a negative undershoot of the impulse response can be recorded, depending on the concentration of calcium ions. They were able to convert an occasional biphasic response to monophasic by lowering the extracellular calcium concentration. The process of chopping/mincing the retina to provide samples for the recording process could also be a factor in disrupting the calcium equilibrium.

Stimulus attributes that control psychophysical detection of flicker closely match those that activate parasol ganglion cells [99,100]. Among the many subclasses of ganglion cells [101–103] there might well be one that can generate a sustained response to an ultra-brief flash of light. However, it seems more likely that sustained retinal activity from such a flash would be driven by the impulse responses of photoreceptors. Ganglion cells generate only a single spike when activated by a single electrical pulse, but produce a train of spikes similar to that elicited by a light flash if the pulse is applied to the neural tissue that lies ahead of the ganglion cells [104]. Additionally, the various psychophysics laws that specify flash effectiveness, such as the Talbot-Plateau law discussed above, argue for an extremely fundamental and stable relationship between intensity and flash salience. That kind of stability is most likely provided at a very early stage in the transduction process. At this point the most parsimonious hypothesis is that impulse responses of photoreceptors service those laws.

Earlier research established that individual letters were recognized using display conditions similar to those provided in the present work [9]. One might assume that if letters can be identified, then so can combinations of letters, *e*.*g*., words and sentences. The present results support this assumption, but it was by no means a foregone conclusion. A number of laboratories have found reaction-time differences as a function of word length, which suggests some degree of sequential processing of letters and/or letter groups [105–107]. The ultra-brief flashes used here provided displays wherein essentially all of the subsequent neuronal activity was post-stimulus, *i*.*e*., based on persistence. Although persistence that is visible lasts for roughly 100 ms (as detailed above), the strength of the stimulus begins to decline immediately, which could impair sequential processing of the letters.

One might have predicted that decay of persistence with the fused-flicker display would have produced an even greater impairment of sentence reading. At 24 Hz, the interval between successive flashes was a bit more than 40 ms, which would be enough for a significant decline in stimulus salience. Early work by Eriksen & Collins [108] found a decline in trigram recognition of roughly 20% due to decay of persistence across a 40 ms interval (drawn from their Fig 2). A 40 ms interval between successive flashes produced a 35% decline in letter recognition in one of Greene & Visani’s experiments (Fig 4 in [9]). Prior to the present work we had no basis for assuming that persistence across a 40 ms interval of darkness would adequately support normal reading.

Based on present results, we can now affirm that the stimulus energy provided by ultra-brief flashes having durations as brief as 1 microsecond can generate persistence of visibility that allows for recognition of words. If the flashes are delivered as a flicker-fused sequence with an intensity meeting the specifications of the Talbot-Plateau law, they drive perceptual processing that is equivalent to a steady stimulus. There appears to be no impairment or handicap of higher-order mechanisms.

## Supporting Information

S1 Methods Additional details are provided on display board and stimulus attributes, ambient illumination, and task demands. (DOCX)

S1 Table Inventory of five-letter words. The words displayed in each of the five experiments are listed.
(DOCX)

S2 Table Inventory of sentences. The sentences that were read in Experiment 5 are provided.
(DOCX)

S1 Fig Distribution of sentence lengths. Sentence length was varied such that the count of letters was distributed as a Gaussian.
(PDF)

S2 Fig Distribution of word lengths. This figure plots the frequency of word lengths tallied across all sentences.
(PDF)

S1 Dataset This folder has five Excel files, each containing the raw data from eight respondents. For Exps 1 and 3 the treatment conditions for a given respondent are shown on successive rows, and the cell entries within the row indicates whether the word was identified (1) or not (0) on the trials wherein that treatment was used. Intensity is specified in machine code (see S1 Methods for conversion). For Exp 2 the respondent reported whether the flash sequence appeared to be fused, i.e., non-flickering; a flicker judgment was scored as 1 and a fused judgment was scored as 0. For Exp 4 the treatment of interest was a brightness judgment; with 1 and 0 indicating whether the two displays were judged as having the same or different brightness, respectively. Exp 5 provides reading time, length of sentence, and reading time as a function of length for each sentence in the S2 Table inventory, each being displayed under fused-flicker or steady display conditions.
(ZIP)
