# Supplementary material for: Recognizing Words and Reading Sentences with Microsecond Flash Displays
Source: PLoS One. 2016 Jan 22;11(1):e0145697. doi: 10.1371/journal.pone.0145697 (PMC4723150; doi:10.1371/journal.pone.0145697)
Supplement: S1 Methods — (DOCX) [file pone.0145697.s001.docx]

**S1 METHODS**

***Stimulus Display Board***

Stimulus displays were provided by a 64x64 array of AlGaInP LEDs (RL5-R8030; Super Bright LEDs, Inc.) that emit at a peak wavelength of 630 nm (red). LED diameters and center-to-center spacing, as seen from a distance of 3.5 m, was 4.91 and 9.23 arc minutes (arc’) of visual angle, respectively. The full span of the array, both horizontal and vertical, was 9.8 degrees of visual angle.

Light emission was controlled by a Propox MMnet101 microcontroller running at a clock speed of 16 Mhz, under program instruction by a Mac Cube computer.

***Display of Words and Sentences***

The individual letters used to compose the words for these experiments were constructed as “thin strokes,” meaning that a string of adjacent dots represented the letter contours.

The five-letter words used in Experiments 1-4 were drawn from a list of 320 that was derived from the standard English dictionary of common words provided on Unix computer systems. An inventory of these words is listed in S1Table.

Experiment 5 used an inventory of 200 declarative sentences that were chosen from Psychology textbooks and the Wall Street Journal (news, entertainment, business, and editorial sections). A listing of the sentences can be found in S2 Table. The selection process rejected any sentence containing a proper noun, punctuation, a parenthetical phrase, or other factors that could make it difficult to interpret what was being said or otherwise impair smooth reading. Sentence lengths were selected to follow a normal distribution with a practical constraint to a minimum of 8 and maximum of 125 letters (see S1 Fig).

Word length varied from 1 to 14 letters with a mean of 4.9 letters. S2 Fig shows a distribution of word lengths. Sentence length ranged from 2 to 26 words (6 to 120 letters), with a mean of 12.4 words (60.5 letters).

Letters were 5 dots tall with a width that ranged from 1 to 5 dot steps. Specified as visual angle, this corresponds to 42.3 arc’ in height, measured to the extreme edges of the dots, with the widths ranging from 4.9 to 42.3 arc’.

Five letter words spanned the same height as individual letters, i.e., 42.3 arc’. Minimum width was 145 arc’ and the maximum width was 239 arc’.

Sentence words had a minimum width of 4.9 arc’ (for the letter A) and a maximum width of 639 arc’. Length of individual sentence lines ranged from 304 to 584 arc’. The number of lines required for display of sentences ranged from 1 to 10, providing a display that ranged from a minimum height of 42.3 arc’ to 546 arc’.

***Respondents and Task Demands***

Forty respondents contributed data, eight for each of the five experiments. They were recruited from the USC Department of Psychology Subject Pool. Displays were judged from a distance of 3.5 m, allowing use of both eyes and with correction of vision as each needed.

The general practice of this laboratory is to determine what treatments and treatment levels to use with pilot testing. Once an experimental design was decided upon, the data from every individual who completes the test session is included in the analysis unless the responses are clearly anomalous. Any rejection of data would be reported. For the present series all who volunteered for testing were able to complete the session without difficulty and all their data has been provided.

In each of the first four experiments all the inventory of words was displayed to a given respondent, one word at a time, requiring the respondent to say the word that was displayed. No feedback was provided as to whether the word was correctly named. None of the tasks were speeded, but responses were generally offered without hesitation. The time required for display and response was about 3-5 seconds per word and the test session for a given experiment was usually completed in about 40 minutes.

Experiments 1 and 3 displayed each word as a single flash or as a sequence of flashes, respectively, varying flash intensities. The only task requirement was to name the word that was displayed.

Experiment 2 displayed each word with a sequence of flashes. In addition to saying what word was shown, the respondents also were asked to judge whether they perceived any flicker in the display (Yes = 1; No = 0).

Experiment 4 displayed each word both with steady emission and as a sequence of flashes. In addition to naming the word the respondents were asked whether the two displays appeared equal in brightness.

Experiment 5 displayed full sentences that were read, the dependent measure being reading time. Respondents were informed that this was not a speeded task and they should try to read each sentence carefully and not misspeak or mispronounce any portion of the sentence. Any defect in reading, *e.g.*, incorrect word, mispronunciation, stammer, was recorded as a zero, which meant that the data for the improperly read sentence would not be included in subsequent analysis of reading time.

***Ambient Illumination, Stimulus Timing, and Intensity***

Ambient light level in the test room was adjusted to 10 lux using opaque occluding panels mounted over standard fluorescent fixtures. This allowed the light level to be reduced without changing the color balance of the light. The illuminance was measured with a Tektronix J 1811 photometer.

This display board provides for 1 microsecond (µs) nominal control of flash duration. However, pulse duration extends somewhat longer depending on the intensity of the flash. For the present experiments the duration of emission, measured at half amplitude from oscilloscope traces, was 1.3 µs. This duration will be described as “ultra-brief” throughout the report, but that term may include somewhat longer durations when referring to prior work done in this laboratory.

The diameter of each LED in the array is less than five minutes of visual angle, so it is most appropriate to consider this as a point source. Intensity has been specified using radiometric units, these being measured with a Thorlabs PM100USB radiometer with a S120C calibrated sensor. This instrument measured the intensity of steady emission from the LEDs, after which flashes were scaled on the basis of oscilloscope traces from a fast photodiode using the Thorlabs readings as anchor measures.

***Experiments and Task Protocols***

The amount of voltage applied to the LEDs determined the intensity of the light emission. Once an intensity range to be used was established by pilot work, treatment levels were based on equal increments of the voltage across the range.

In each of the experiments described below, the order in which treatments were administered was random. In each experiment all 26 letters of the alphabet were displayed in a random order for each treatment level.

Experiment 1 presented each word as a single and simultaneous ultra-brief flash of every dot in the letters forming the word. Intensity was varied to provide a single-flash activation curve, *i.e.*, specifying hit rate as a function of flash intensity. Radiant intensity was varied across 16 levels, these being: 564, 708, 853, 1004, 1162, 1320, 1495, 1669, 1844, 2022, 2200, 2382, 2565, 2751, 2939, 3127 µW/sr. Words that were correctly named were scored as one (1) and incorrectly identified words were scored as zero (0). The proportion of words correctly identified at a given intensity is designated as the “hit rate” for that intensity.

Experiment 2 displayed each word as a sequence of ultra-brief flashes, with the frequency of the sequence being varied and the full duration of the sequence being 750 milliseconds. Each and all flashes were delivered with an intensity of 2588 µW/sr, this being the mean of the intensities at which respondents in Experiment 1 first reached their highest hit rate. Frequency was varied from 6 to 30 Hz in increments of 2 Hz, providing 13 treatment levels.

Experiment 3 displayed each word with a sequence of 24 Hz ultra-brief flashes, with the full duration of the sequence being 750 milliseconds. Flash intensity was varied across 13 steps, specifically: 139, 200, 314, 429, 674, 963, 1439, 2043, 2734, 4026, 5518, 7338, 10140 nW/sr. [Note that units are nanowatts/steradian.]

Experiment 4 displayed each word twice, once as a fused-flicker display and also with a steady display, the order of these two treatments being random. Each of the display conditions had a 750 millisecond duration, separated by a 250 millisecond gap. A fixed intensity of 2573 µW/sr was used for all flashes, and steady displays varied the intensity at the following 16 levels: 23, 31, 39, 47, 55, 64, 85, 107, 129, 139, 161, 200, 257, 314, 371, and 429 nW/sr (again, nanowatts per steradians).

For Experiment 4 the comparison of average intensity of flashes with the intensity of steady display was of special interest, for the classic Talbot-Plateau law predicts that they should be equal when the two displays are seen as equally bright. At 24 Hz the average intensity of flashes was calculated to be 86 nW/sr.

Experiment 5 displayed a random sampling from the sentence inventory, each sentence being shown either as a 24 Hz fused-flash sequence or as a steady display, with radiant intensities being 2588 µW/sr and 102 nW/sr, respectively. Sentences were chosen at random from the inventory and assigned to either the fused-flicker or steady condition. If the sentence was read without defect, *i.e.*, without stammering, misreading, or mispronouncing any word, the time required to read the sentence was recorded. This continued until 80 sentences had been acceptably read for each of the display conditions. The experimental protocol had included a requirement that all of the respondent’s data would be rejected if more than 20% of the sentences were misread. The inclusion of a 20% cushion turned out to be overly cautious, as respondents read most of the sentences without defect, the maximum number of errors being 21 sentences – 10.5%.
