# Supplementary material for: Recognizing Words and Reading Sentences with Microsecond Flash Displays
Source: PLoS One. 2016 Jan 22;11(1):e0145697. doi: 10.1371/journal.pone.0145697 (PMC4723150; doi:10.1371/journal.pone.0145697)
Supplement: S1 Table — The words displayed in each of the five experiments are listed. (DOCX) [file pone.0145697.s002.docx]

Supplemental Table 1: Words used for Experiments 1-4

frisk

crawl

helix

crust

flung

polar

flash

snack

nomad

decay

count

knock

slang

below

spurn

tardy

draft

plunk

stole

tease

gismo

shirt

swamp

quiet

macro

murky

chile

spawn

adept

waxen

bongo

quack

grind

diner

other

stern

feast

issue

equip

unzip

elder

mocha

thigh

stick

gecko

plank

rigor

favor

baker

match

shown

stalk

eater

water

guard

crony

might

truth

relax

adobe

axiom

brisk

panty

acorn

churn

awash

whirl

giant

bride

focal

naval

shrew

gorge

taken

bicep

panic

condo

lapse

epoxy

glade

stout

defer

wrung

inert

nylon

latch

sword

cling

chase

kaput

haven

crank

climb

grant

vocal

blond

awful

purse

ranch

ratio

razor

layer

stray

imbed

pasty

pouch

leach

spent

circa

amuse

resin

tempo

ninth

quest

juror

stump

mouth

naked

graze

clown

chomp

human

forum

blaze

older

tipsy

given

fiber

saver

letup

godly

faint

crisp

peace

balky

logic

slush

hitch

spite

clock

blunt

exude

stamp

anvil

awoke

vivid

molar

petal

scary

dream

horse

alien

trump

straw

above

badge

stunt

rider

verse

shift

power

sedan

tonal

ghost

squad

tenth

graft

spend

perch

adorn

whale

toxin

deter

shape

urban

buyer

clash

tract

clung

satin

smirk

birth

knife

ameba

paper

mumps

noisy

ripen

chick

fresh

salve

mirth

quart

rinse

along

stove

smile

clerk

seven

plump

hound

aside

flora

clump

steal

chink

state

weary

dogma

navel

impel

xenon

clean

swept

melon

savor

torch

begin

infer

dense

bound

ether

bring

coast

patio

fixer

began

uncut

glove

depth

laugh

bland

shove

swift

slash

death

prowl

trial

towel

sense

drunk

float

basis

taker

karma

brush

venom

drank

bingo

exalt

fairy

check

loyal

river

snarl

handy

adopt

reach

worth

bribe

glide

sworn

fatal

image

prose

shame

claim

munch

alpha

found

valve

apart

throb

cycle

brave

mousy

toast

motor

stare

stark

evoke

undue

finch

atlas

token

ditch

lotus

mount

enjoy

jerky

japan

juice

joker

junky

jumbo

quote

equal

quirk

quake

squaw

quilt

quick

seize

zebra

glaze

prize

dozen

amaze

froze

craze

excel

exert

toxic

sixth

boxer

banjo

eject

jewel

joint

judge
