# Supplementary material for: Recognizing Words and Reading Sentences with Microsecond Flash Displays
Source: PLoS One. 2016 Jan 22;11(1):e0145697. doi: 10.1371/journal.pone.0145697 (PMC4723150; doi:10.1371/journal.pone.0145697)
Supplement: S2 Table — The sentences that were read in Experiment 5 are provided. (DOCX) [file pone.0145697.s003.docx]

Supplemental Table 2: Sentences displayed in Experiment 5

Movies often get by on style.

The movie itself runs a little slow and flat.

We wanted to honor the greatest car movies of all time

People ask me to call them on a daily basis.

There is very little story in a videogame but there is a format.

Here are your best job options.

The mayor is a small and politically vicious man.

Outsized gains in stock prices relative to property values mean that wealth is even more concentrated at the top.

Wealth gains that go to the richest Americans do not power spending in the same way as when they are more evenly spread.

The recovery in home prices may not power spending in the way that rising prices did before the crash.

It is probably a good thing that homeowners no longer think of their residence as a source of ready cash.

These are schools that work.

On Thursday the Mayor went on a sympathetic radio station and could not have been more clear about what drove him to this course of action.

Having products appear in a program has been a part of the television business since the early days of the medium.

Product placement has become more popular as marketers look for new ways to free themselves from commercial breaks.

Skipping ads is far less common during an event like the Academy Awards.

The lines between entertainment and advertising continue to blur and experts warn that these gimmicks could turn off buyers.

Many experts claim that wages have fallen behind productivity gains over the last generation.

Fringe benefits make up a larger share of the typical paycheck than they did forty years ago.

Different inflation adjustments give conflicting estimates of just how much the purchasing power of the dollar has fallen.

Membership in the middle class seems to be declining but this is because more American households are moving up.

The competition for labor services is what links pay to productivity.

They move in tandem.

University of California officials say they are still struggling to meet diversity goals.

There were many internal debates that shaped the law.

Doctors would be paid one fee for the entire episode of care regardless of how many tests were needed.

We considered the idea of phasing in bundled payments.

But then we hit a brick wall.

This was a far cry from the ambitious plan that many of us hoped for.

Understanding the human mind is the most exciting scientific challenge of our time.

Great progress has been made in unraveling the relationship between mind and brain.

These relationships are best understood in the realm of perception.

Our understanding of how we perceive the world has undergone a revolution in the past two decades.

My aim is to tell a convincing story.

Each chapter will include the following features.

Chapter one lays out the foundations of research in sensation and perception.

It provides basic information on how neurons convey information within vast networks.

The experiments discussed throughout the rest of the book all employ one or more of these methods.

He awoke and felt a sharp and unfamiliar pain.

He was having a stroke.

Many students are surprised to learn that the field of psychology has been around only for a century.

This created more problems than it solved.

The relationships among the elements as a whole determine our experience.

Computers and people store information and must have mechanisms for doing so.

Throughout the book we will review the various research methods that were used.

She is a name to watch out for in the coming season.

Every year exceptional actors are honored for their performances in film.

This person is fearless and can engage at the highest levels.

We watch with excitement as the stars show up to receive their golden statues.

Most salespeople have a secret desire to be actors but sales are a much faster way to make money.

We give the most persuasive presentations at the end of the sales year.

We read and respond to body language and use the power of our voices.

Take a minute and consider who deserves recognition.

She knows how to move the sale along.

Everyone calls him before they call technical support.

A recent study discovered that even employees who are well focused don't respond to negative performance reviews.

Employees are more accepting of feedback when the source is credible.

The strategies of these two businesses are quite different.

There are numerous benefits of performance reviews.

Make it a conversation that matters rather than an event that all parties dread.

The best thing is to always take action and be exposed to opportunities.

It is a special thing to find people with the same mindset and feel this community around you.

The team has only a few days to decide whether to keep working on the project.

The right equipment is the key to making it across the finish line.

Many of them compressed their training regimen with the help of a coach.

Some gear is designed to shave off weight.

It is not uncommon for athletes to have their training sessions scientifically calibrated.

The investment is now paying off as Americans renew their relationship with paper.

There is no question that paper companies have suffered over the past ten years.

Efforts by the government to go paperless landed the earliest blows.

Noise cancelling headphones have not been as popular as one would think.

The technology is here whether you like it or not.

At times it would sound garbled to the person at the other end.

They will say that I never actually played as a child.

From a very young age I liked to organize the toys in my room.

The games children plan are a window into their minds.

The Latino community in my childhood had clear expectations for each gender.

My brothers and I used to play war with the other kids in the neighborhood.

Fortunately we saw our mother break this mold by running for our local school board.

Social scientists have long studied how language affects society.

Berlin is a real city that never sleeps.

Two new lodging options provide that and even more.

The two and three bedroom suites feature fully stocked kitchens and bars.

Online programs offer veterans and their spouses career assistance adapting to corporate life.

The exposed brick walls are adorned with constantly changing art exhibitions.

The eatery serves regional food presented with supreme polish.

Getting a dinner reservation can be tricky.

One thing I do not have is a computer science degree.

Online programs offer veterans and their spouses career assistance adapting to corporate life.

The program provides career counseling and classes for soldiers and their families.

Do not limit yourself to stereotypes.

Another network theory of memory has been developed and refined over several years.

He believed that declarative memory stores information in networks that contain nodes.

The first question that will concern us is how stored knowledge is organized.

There are several distinct ways of arranging and storing information.

Suppose you want to find a particular book but have forgotten its name.

Consider this real life example of medical diagnosis.

Many models have been developed in the field of artificial intelligence.

You have stored a great deal of knowledge.

Physicians are not the only ones who categorize illnesses.

The study of visual imagery has a controversial history.

Interest in visual imagery never completely vanished.

You likely know that the state of California is west of the state of Nevada.

This is a true fact.

Other investigators have argued for a different proposal.

We dissect nature along lines laid down by our native language.

Certain rules can be used to draw valid conclusions from the data.

All of us engage in inductive reasoning.

Very few commonly heard sounds approximate pure tones.

The quality of the sound depends on the frequency and amplitude.

A better method is to ask people to say when two tones seem equally loud.

This experiment is performed for all frequencies.

Addressing all these questions will require further investigation.

Our brains have evolved to perform such calculations.

Professional baseball pitchers routinely throw a fastball at ninety miles per hour.

The batter cannot track quickly enough to follow the path of the fastball.

This means that the ball must be hit when it is well outside the center of gaze.

Part of the answer is that the batter does not wait until the pitch is released.

Daylight savings time arrived this weekend in the United States.

Most people will set their clocks sixty minutes ahead before going to bed.

Forestry officials have called in some experts.

This kind of memory refers to the initial brief storage of sensory information.

Rehearsal is thought to help the items enter long term storage.

The recency effect is thought to result from using either sensory memory or short term memory.

It is thought that incoming sensations first pass through a rapidly decaying information storage system.

Other work investigated how many ways participants could be cued to give partial reports.

There is also a memory for auditory material.

No time now.

Not now.

This suggests that the information in the icon is visual rather than auditory.

In one condition the subjects were asked to report all the letters that were present.

The persistence with which this question plagues me is much more than an inconvenience.

Most people want to hold on to the information more than a few moments.

He demonstrated that if you are shown a string of random numbers you will recall only a few.

Someone not familiar with our culture might regard these as completely random letters.

The number has assumed a variety of disguises that are sometimes a little larger and sometimes smaller.

You first learn a list of French words by pairing them with their English counterparts.

Some researchers believe that interference plays a role in most forgetting of facts from long term memory.

European governments have taken remarkable strides toward more integration over the past four years.

Polls suggest that voters are unimpressed.

All of this would have been unthinkable before the current crisis.

Even in rural France the support stands as sixty three percent.

This reflects the intentions of the old voters.

Opposition to the European Union is being fueled by anxieties over immigration.

The struggle for long term growth will increasingly become a political rather than a financial problem.

For many this habit may be too ingrained to change tactics now.

The mystery over what happened to the airplane has baffled investigators and airline officials for much of the weekend.

The government said that it could not land a seaplane near the objects to investigate them further because it was getting too dark.

Those on the chase encountered what appeared to be decoys that seemed designed to put them off the scent.

The virtual currency remained a niche hobby until its price jumped and mainstream companies began accepting it as payment.

The magazine says that it stands by the story.

One message appeared late Thursday on an internet message board.

The poll from earlier this year showed that many of the people working in these offices are dissatisfied about their current work situation.

It became extremely clear that innovative use of technology is important in helping improve productivity.

Financial advisers as a group are traditionally wary of management changes in their highly competitive industry.

Advisers are nervous about changes that might signal greater control by banking executives.

The managers were pushed out.

At least one manager apparently left without a new job lined up.

Departures and retirements are not unusual early in the year.

You are sitting at your desk trying to make something out of nothing.

They would flash through all the reasons to hang up.

Clients will not follow just because we get a better deal somewhere else.

Most days he tried to be polite.

On some level the calls are flattering.

It is nice when somebody other than the mother ship notices your business.

You should use the recruiter questions to shed light on your business and cultivate long term allies.

These relationships turn to pure gold when it is time to jump ship.

Traces of four previously undetected man-made gasses have been discovered in the atmosphere.

The ozone layer is vulnerable to a range of chemicals including those once commonly used in refrigerators.

They do not know how and where these gasses are being emitted.

More than two dozen veteran managers have quietly retired in recent weeks.

On average the departing managers spent more than twenty years on the job.

A saleswoman played down the changes.

Another advisor also called the changes truly surprising.

The use of stolen passports by two passengers raised concerns over airport security.

Law enforcement officials say the stolen passports would have been detected during checkin.

She wanted a bookstore of her own.

Her attempts to get a bank loan failed.

A bookstore in her hometown came up for sale.

Her experience illustrates a common challenge for first time entrepreneurs.

Securing a bank loan can be difficult proposition no matter the state of the economy.

Banks think of startup ventures as being especially risky.

Wireless carriers have adjusted their prices again in an aggressive push for customers.

The new plan would lower the cost for a subscriber who bring or buys his own phone.

The company began aggressively adding customers last year by doing away with contracts.

Regulators praised the company for changing the contract.

Others in the industry have questioned how long they can afford to keep up the pressure.

It has been merely a month since Congress passed a one trillion dollar stimulus bill.

The television series is counting on positive viewer reactions as the show returns for its fifth season.

They prefer to stack the deck with layers of story lines to fend off predictability.

He found inspiration by meditating on the quest to understand the scientific world.

To be able to hold onto that even a little bit through your life is a great starting point.

The surge has helped this sector bounce back from the bottom of the economic crisis.

Other factors included an improving economy and the proximity to ports.

A lot of retailers tried to respond to demand and see how they can deliver their product a lot quicker.

The warehouses are almost all leased and developers have turned to speculative building.

When they take standardized examinations the students in charter schools often outscore their counterparts at conventional public schools.

Building of sprawling shopping centers slowed for many years after the economic crisis.

This is no time for the faint of heart to enter such a difficult and volatile area of business.

Cooler heads prevailed when they saw there was little reason to continue to escalate their fight.
