# Supplementary figures and images for: Recognizing Words and Reading Sentences with Microsecond Flash Displays
Source: PLoS One. 2016 Jan 22;11(1):e0145697. doi: 10.1371/journal.pone.0145697 (PMC4723150; doi:10.1371/journal.pone.0145697)

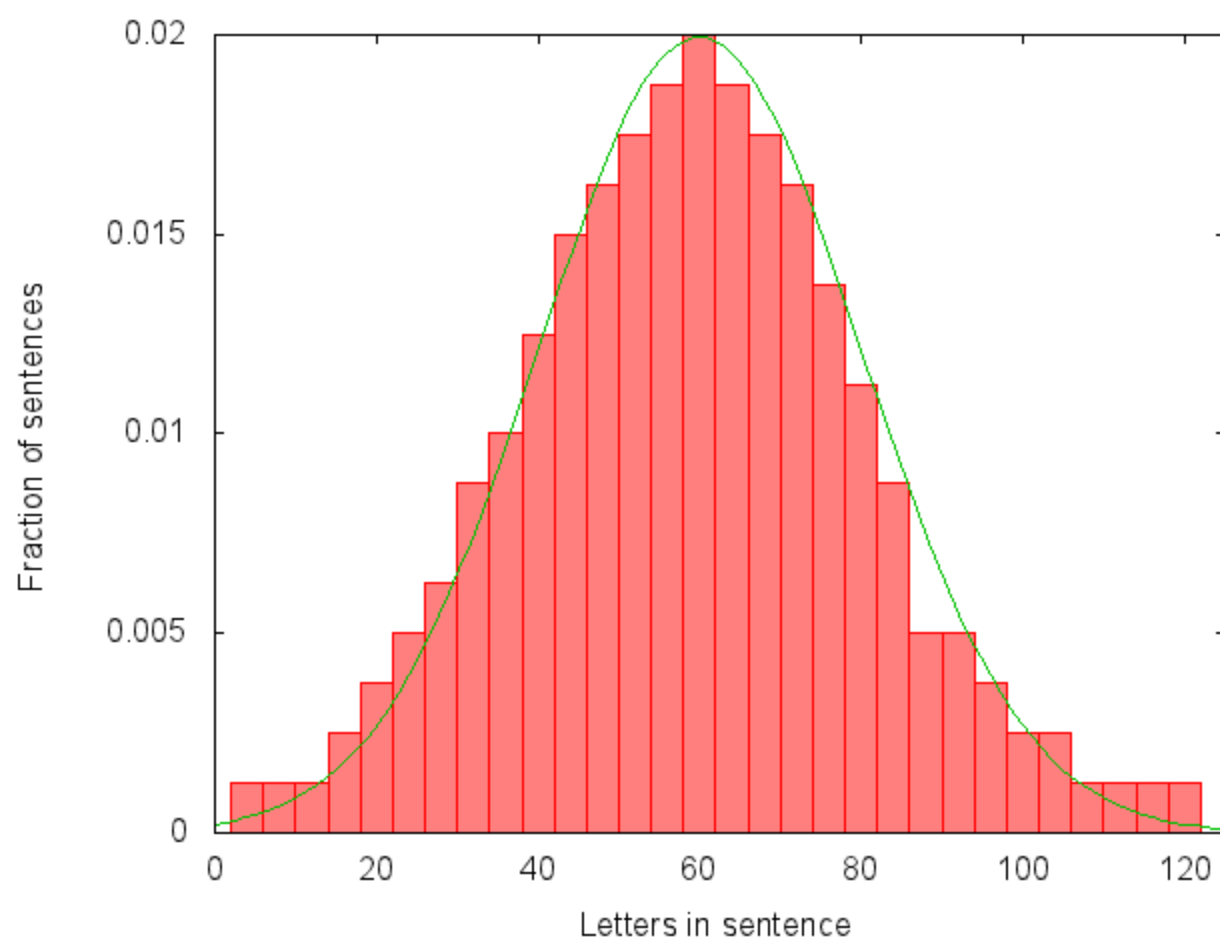

Supplement: S1 Fig — Sentence length was varied such that the count of letters was distributed as a Gaussian. (PDF) [file pone.0145697.s004.pdf]

Frequency

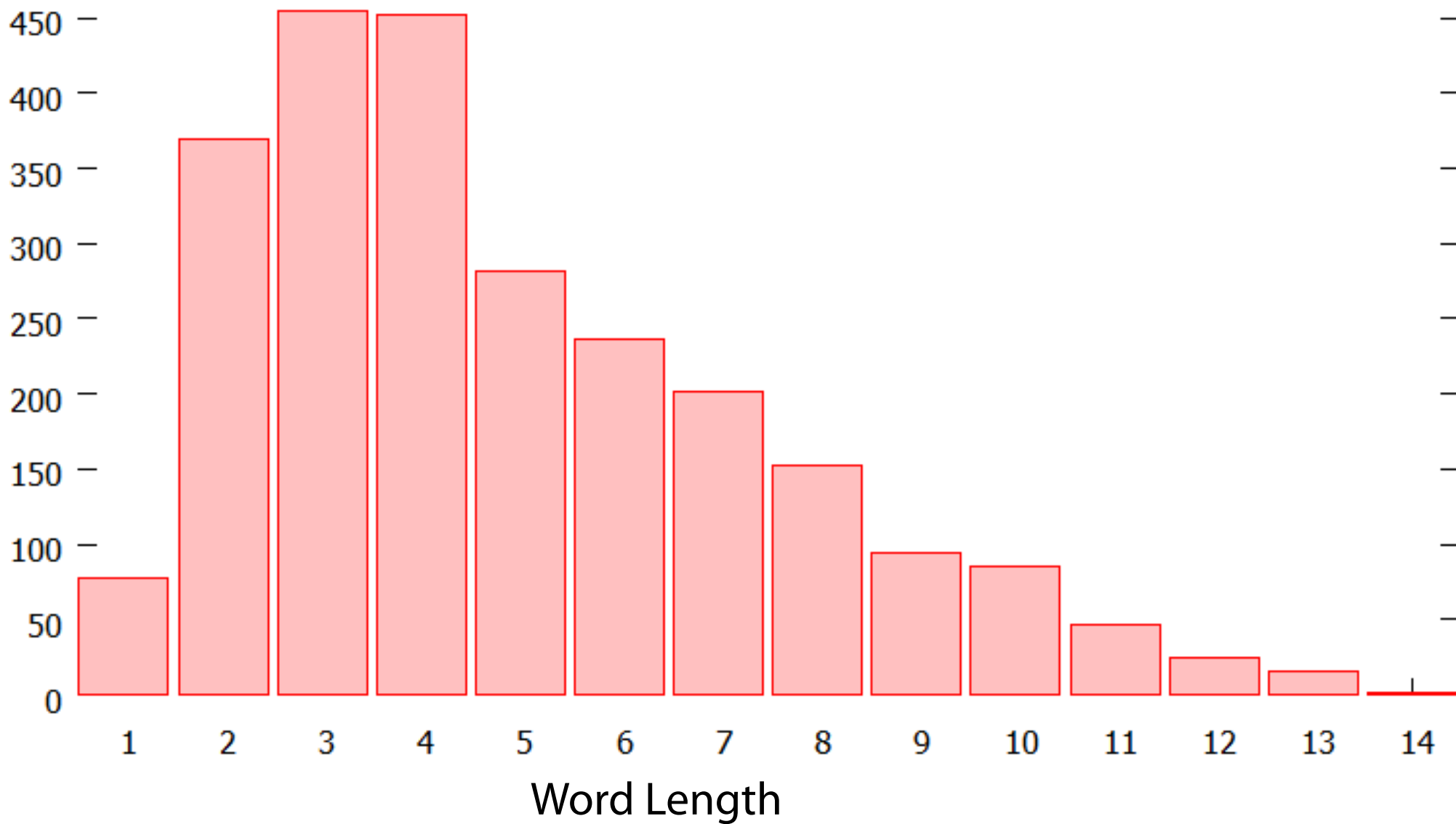

Supplement: S2 Fig — This figure plots the frequency of word lengths tallied across all sentences. (PDF) [file pone.0145697.s005.pdf]
